# Population Dynamics of *Phytophthora infestans* in the Netherlands Reveals Expansion and Spread of Dominant Clonal Lineages and Virulence in Sexual Offspring

**DOI:** 10.1534/g3.112.004150

**Published:** 2012-12-01

**Authors:** Y. Li, T. A. J. van der Lee, A. Evenhuis, G. B. M. van den Bosch, P. J. van Bekkum, M. G. Förch, M. P. E van Gent-Pelzer, H. M. G. van Raaij, E. Jacobsen, S. W. Huang, F. Govers, V. G. A. A. Vleeshouwers, G. J. T. Kessel

**Affiliations:** * Institute of Vegetables and Flowers, Chinese Academy of Agricultural Sciences, 100081, Beijing, China; † Bio-interactions and Plant Health, Plant Research International, 6700 AA Wageningen, The Netherlands; ‡ Laboratory of Plant Breeding, Wageningen University, 6708 PB Wageningen, The Netherlands; § Laboratory of Phytopathology, Wageningen University, 6700 EE Wageningen, The Netherlands; ** Centre for BioSystems Biology, 6700 AB Wageningen, The Netherlands

**Keywords:** late blight, Blue_13, avirulence, microsatellites, population genetics

## Abstract

For a comprehensive survey of the structure and dynamics of the Dutch *Phytophthora infestans* population, 652 *P. infestans* isolates were collected from commercial potato fields in the Netherlands during the 10-year period 2000–2009. Genotyping was performed using 12 highly informative microsatellite markers and mitochondrial haplotypes. In addition, for each isolate, the mating type was determined. STRUCTURE analysis grouped the 322 identified genotypes in three clusters. Cluster 1 consists of a single clonal lineage NL-001, known as “Blue_13”; all isolates in this cluster have the A2 mating type and the Ia mitochondrial haplotype. Clusters 2 and 3 display a more elaborate substructure containing many unique genotypes. In Cluster 3, several distinct clonal lineages were also identified. This survey witnesses that the Dutch population underwent dramatic changes in the 10 years under study. The most notable change was the emergence and spread of A2 mating type strain NL-001 (or “Blue_13”). The results emphasize the importance of the sexual cycle in generating genetic diversity and the importance of the asexual cycle as the propagation and dispersal mechanism for successful genotypes. Isolates were also screened for absence of the *Avrblb1/ipiO* class I gene, which is indicative for virulence on *Rpi-blb1*. This is also the first report of *Rpi-blb1* breakers in the Netherlands. Superimposing the virulence screening on the SSR genetic backbone indicates that lack the *Avrblb1/ipiO* class I gene only occurred in sexual progeny. So far, the asexual spread of the virulent isolates identified has been limited.

The oomycete *Phytophthora infestans* is the causal organism of late blight on potato and tomato. Globally, late blight carries multiple costs, including complete crop failures, economic losses due to decreased yields, and fungicide applications with a potentially negative impact on human health and the environment (Forbes 2011). In the Netherlands, the total area under potato cultivation amounts to 165,000 hectares and annually yields 7.9 million tons of potato, representing a farmgate value of about 790M€. The number of fungicide applications varies between 10 and 16 per season. Costs for potato late blight control (chemicals, application, and losses) amount to 125M€ per year, almost 16% of the total farmgate value (Haverkort *et al.* 2008).

From these figures, it is clear that farmers, the potato industry, consumers, and the environment could greatly benefit from more efficient and environmentally friendly ways to control late blight through, for example, the introduction and durable exploitation of host plant resistance. *P. infestans*, however, is renowned for its capacity for adaptation, particularly with respect to emergence of virulence toward resistant cultivars and, to a lesser extent, fungicide resistance (Haas *et al.* 2009). One of the prerequisites for durable management of late blight, therefore, is up-to-date knowledge on characteristics of the local *P. infestans* population and high-level understanding of population dynamics in order to avoid erosion of cultivar resistance and development of fungicide resistance. Currently, one of the most promising *R* genes is the broad-spectrum resistance gene *Rpi-blb1* (Song *et al.* 2003; van der Vossen *et al.* 2003), which is used in breeding programs.

*P. infestans* is heterothallic, and both A1 and A2 mating types are required for completion of the sexual cycle. Sexual reproduction results in high levels of genetic variation in the offspring and may lead to increased and more rapid evolution of the pathogen. In the Netherlands, only the A1 mating type was found prior to the 1980s, and all isolates were grouped in a single (US1) clonal lineage (Drenth *et al.* 1994), which was also found in many other parts of the world (Drenth *et al.* 1993). During the 1980s, following a renewed global migration of both (A1 and A2) mating types, a new *P. infestans* population rapidly displaced the US1 clonal lineage (Drenth *et al.* 1993; Spielman *et al.* 1991) in the Netherlands. Isolates having the US1 genotype have not been detected in the Netherlands since (Drenth *et al.* 1994; Spielman *et al.* 1991). One of the driving forces behind this displacement may have been the higher levels of aggressiveness and fitness in the new population compared with the old population (Flier and Turkensteen 1999). The newly introduced *P. infestans* genotypes in combination with the occurrence of sexual reproduction also considerably raised the level of genetic diversity in the Dutch *P. infestans* population, leading to a highly variable population (Drenth *et al.* 1994) with a presumed higher level of adaptability compared with the previous, purely asexually reproducing population.

More than two decades after the introduction of new genotypes in Europe, investigators from the UK reported that a single *P. infestans* genotype with A2 mating type, EU13_A2 (or “Blue_13”) is dominant in the UK (Cooke *et al.* 2012). The dominant position of “Blue_13” was hypothesized to have emerged from superior levels of fitness in combination with resistance to the frequently used metalaxyl and a “Blue_13” favorable choice of commonly grown cultivars (White and Shaw 2009).

Molecular markers provide the opportunity to track and trace individual genotypes and to study population diversity. In the past, genotypic characterization of *P. infestans* isolates included allozyme pattern, mitochondrial (mt) DNA haplotype, RG57 RFLP fingerprints, and AFLP fingerprinting (Drenth *et al.* 1994; Fry *et al.* 1991, 1992; Spielman *et al.* 1991). Co-dominant markers such as microsatellites, also known as simple sequence repeats (SSR), have previously been used to investigate the genetic structure and reproductive biology of numerous plant pathogens (Tenzer *et al.* 1999). Particularly for diploid or aneuploid species such as *P. infestans*, SSRs may provide a better understanding of the overall genetic structure. A recently developed and standardized set of 12 highly informative microsatellite markers (Knapova and Gisi 2002; Lees *et al.* 2006; Li *et al.* 2010) was exploited to perform high-resolution genotyping. In addition, we developed a new assay to monitor for virulence on *Rpi-blb1* (van der Vossen *et al.* 2003), also known as *RB* (Song *et al.* 2003). The corresponding avirulence gene *Avrblb1*/*ipiO* class I (Clark and Jasieniuk 2011; Pieterse *et al.* 1994), is genetically highly diverse in *P. infestans*. Absence of *Avrblb1*/*ipiO* class I has been reported to be associated with virulence on potato plants carrying *Rpi-blb1* (Champouret *et al.* 2009). In this study, we combine genotyping with both functional and neutral markers.

On the premise that understanding of the population genetics of plant pathogens will contribute to the development of more sustainable disease management strategies, the objective of this study was to describe and analyze the dynamics of the Dutch *P. infestans* population over the course of the decade between 2000 and 2009. Four major topics were addressed: (i) the distribution of genetic variation within the Dutch population associated with geographical levels (national *vs.* regional *vs.* local populations); (ii) a description of the occurrence and dynamics of dominant clonal genotypes in the Netherlands; (iii) monitoring for the virulence for the *Rpi-blb1* gene; and (iv) the origin of isolates virulent on *Rpi-blb1*.

## Materials and Methods

### Isolate sampling

**Figure 1:**
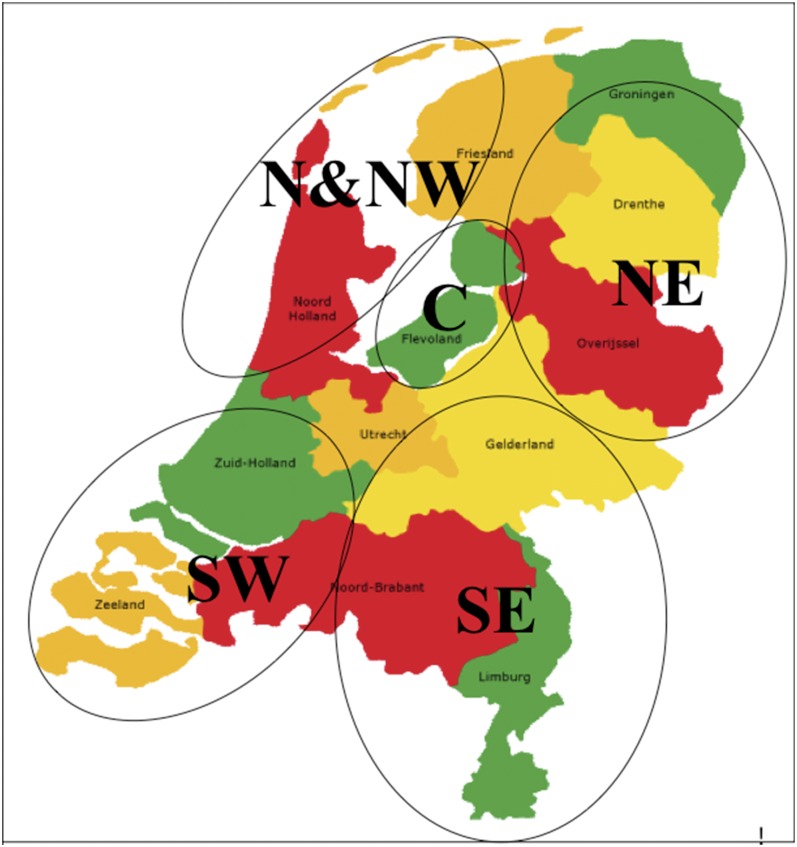
Map of the Netherlands showing the regions where the *P. infestans* isolates analyzed in this study were sampled.

Isolates were collected from 207 different locations (Table 1) encompassing all five major Dutch potato-growing areas (Figure 1). A total of 676 *P. infestans* isolates was analyzed in the genetic clustering, including 652 isolates from the 2000–2009 samplings and 16 isolates from the 1980s and 1990s collection representing older populations, and 8 isolates used as reference for the SSR markers.

**Table 1:** Total isolates and “Blue-13” isolates from different years and sampling regions

Region	2000	2001	2002	2003	2004	2005	2006	2007	2008	2009	Sum
C	8	0	4	10	6	34/25*^a^*	28/16	16/12	10/5	34/6	150/64
N & NW	6	1	0	4	2	23/14	6/3	9/5	4/4	0	55/26
NE	18	22	20	21	28/4	30/6	18/4	23/11	9/7	20/3	209/35
SE	19	1	2	7	17/5	19/15	10/6	16/10	24/13	23/16	138/65
SW	4	0	3	10	3	46/5	10/4	16/12	2/2	6/6	100/29

a The second number indicates the number of “Blue-13” isolates.

Sampling areas (Figure 1) were categorized according to geographical location and type of potato cultivation. The North East (NE) of the Netherlands is characterized by starch potato crops grown on sandy and peat soils. The North (N), North West (NW) and South West (SW), as well as the Central (C) areas are dominated by ware and seed potatoes grown on clay soils. The regions N and NW are close to each other and therefore treated as one region (N & NW) in this study. The South East (SE) is characterized by ware potato crops on sandy soils.

Production fields with potato late blight outbreaks were reported by the extension services of private companies (Cebeco Agrochemie, Agrifirm, CropSolutions, Agrarische Unie, Profyto, Nestlé, Syngenta, Bayer, HLB, DLV, Dacom, and Agrovision) and potato growers. Infected leaves were predominantly sampled from production fields but also from allotment gardens, potato dumps, and volunteer potato plants. The samples were collected during the whole growing season, generally between the first of April and the end of September. Location, sampling date, and if known, cultivars were recorded. A maximum of two samples per field was collected. Samples were preferably taken from distinct foci and never from the same plant.

### Isolation of *P. infestans*

Infected potato leaflets containing a single lesion were handpicked and positioned upside down in a Petri dish containing 1.5% water agar. Petri dishes were closed, sealed, and sent to the laboratory. A small tissue sample from the edge of the lesion was then placed under a potato tuber disc (approximately 0.5 cm thick) inside an otherwise empty Petri dish and incubated for one week at 15° at a light intensity of 12 Wm^−2^ for 16 hr. Mycelium emerging from the top of the tuber slice was then transferred to ampicillin-contained (1.6 mg/L) pea agar (PA) (Goodwin *et al.* 1992). All isolates (Table 1 and Supporting Information, File S1) were stored in liquid nitrogen and maintained as part of the *P. infestans* collection at Plant Research International in the Netherlands.

### Determination of mating types

An agar plug, obtained from the edge of an actively growing colony of the isolate, was transferred to PA on one side of the Petri dish. A similar mycelium plug of an A1 (isolate VK98014) or A2 (isolate EC3425) tester isolate was placed on the other side. Plates were incubated in the dark for 14–21 days at 18°. After mycelial contact between both colonies was established, the contact zone was monitored for the presence of oospores during 7 days using a microscope at 100× magnification. When oospores were found in the Petri dish with the A1 tester isolate, the unknown isolate was classified to have the A2 mating type and vice versa.

### DNA extraction

Agar plugs of the individual *P. infestans* isolates, which were taken from the edge of a 7-day-old, actively growing colony on PA, were transferred to liquid pea broth. After 3–4 days of incubation at 20° in the dark, mycelium was collected for lyophilization and subsequent DNA extraction. Genomic DNA was isolated from 20 mg of lyophilized mycelium using the DNeasy 96 Plant Kit (Qiagen, Hilden, Germany). The procedure followed the detailed manufacturer’s instructions. Elution was done with 200 µl ultra-pure water. DNA extracts were stored at −20° until further use.

### Haplotype test

Mt haplotypes were determined using the PCR-RFLP method of Griffith and Shaw (1998). Restriction digestions of the amplified regions P2 (*Msp*I) and P4 (*Eco*RI) allowed for differentiation of the four mt haplotypes Ia, Ib, IIa, and IIb.

### SSR amplification

Twelve microsatellite markers were used (Table 2) (Knapova and Gisi 2002; Lees *et al.* 2006; Li *et al.* 2010). Amplification of the SSR markers was carried out as described by Li *et al.* (2010). To facilitate scoring and data entry in the international Euroblight database, eight reference isolates were used (T30-4, 80029, 88133, VK1.4, 90128, IPO-0, IPO428-2, and VK98014). PCR volume (5 µl) was diluted 1000 times, of which 1 μl was added to 9 μl of deionized formamide containing 0.045μl of GeneScan-500LIZ standard (Applied Biosystems, USA). The mixture was capillary electrophoresed on an automated ABI 3730 according to the manufacturer’s instructions. SSR allele sizing was performed using GeneMapper version 3.7 software (Applied Biosystems).

**Table 2:** Microsatellite polymorphism across loci and regions

		D13	G11	Pi04	Pi4B	Pi63	Pi70	PinfSSR2	PinfSSR3	PinfSSR4	PinfSSR6	PinfSSR8	PinfSSR11	Mean
	Dye color	FAM	NED	VIC	PET	VIC	VIC	PET	NED	FAM	VIC	FAM	NED	
	Size range	100–185	130–180	160–175	200–295	265–280	185–205	165–180	255–275	280–305	230–250	250–275	325–360	
	PIC value	0.536	0.841	0.632	0.592	0.560	0.128	0.377	0.533	0.668	0.538	0.576	0.430	0.534
	Frequency of rare alleles	0.429	0.143	0.036	0.000	0.036	0.000	0.036	0.107	0.179	0.000	0.036	0.000	0.084
	Na	17	13	4	3	4	3	3	6	11	3	4	4	6.25
	Ne	1.872	6.151	2.722	2.447	2.218	1.155	1.614	2.086	2.733	2.120	2.364	1.752	2.436
	I	1.207	2.006	1.061	0.993	0.935	0.277	0.586	0.894	1.243	0.899	0.983	0.779	0.989
	Ho	0.209	0.662	0.772	0.766	0.720	0.114	0.419	0.713	0.809	0.638	0.671	0.436	0.577
	He	0.466	0.838	0.633	0.592	0.550	0.134	0.381	0.521	0.635	0.529	0.578	0.429	0.524
	*P**^a^*	1.000	1.000	0.972	0.000	0.000	0.993	0.005	0.000	0.008	0.000	0.843	0.176	
	*P* (clone corrected)*^a^*	1.000	1.000	0.99	0.000	0.000	0.993	0.001	0.000	0.442	0.000	1.000	0.098	
	Fst	0.018	0.023	0.075	0.016	0.014	0.006	0.012	0.006	0.003	0.011	0.000	0.025	
C	L3	10/151	9/151	0/151	1/151	6/151	0/151	0/151	5/151	16/151	3/151	1/151	4/151	
N&NW	L3	7/55	1/55	0/55	0/55	1/55	0/55	0/55	2/55	3/55	0/55	0/55	0/55	
NE	L3	12/209	4/209	0/209	0/209	0/209	0/209	0/209	1/209	7/209	4/209	0/209	0/209	
SE	L3	7/138	5/138	1/138	1/138	0/138	0/138	0/138	5/138	21/138	3/138	0/138	0/138	
SW	L3	1/100	10/100	0/100	0/100	1/100	0/100	0/100	9/100	17/100	6/100	0/100	0/100	

PIC, polymorphism information content; Na, observed number of alleles; Ne, effective number of alleles; I, Shannon\x{2019}s information index (Lewontin, 1972); Ho, observed heterozygote; He, expected heterozygote; *P*, *P*-value; Fst, fixation index; L3, number of individual isolates having the locus with three alleles; the letters in the bottom of the first column are the abbreviations of the regions (see *Materials and Methods*).

a Significant *P* < 0.05 deviation of HWE; the deviation after Bonferroni correction is the same as before.

### Genetic data analysis

#### Clone correction:

To eliminate the bias imposed by the large asexual reproductive capacity and to avoid redundancy in the collection (Chen and McDonald 1996; Kumar *et al.* 1999), a “clone-corrected dataset” was constructed by including only one representative isolate of each genotype. Thus, a data subset containing 358 isolates remained. This data subset was used for the analysis of the Hardy-Weinberg equilibrium (HWE), linkage disequilibrium (LD) and served as input to the STRUCTURE software and the principal coordinate analysis (PCA). The full dataset containing 652 isolates was used for analysis of genetic diversity and distance-based clustering.

#### Ploidy level:

In analyzing the variation in microsatellite loci, most *P. infestans* isolates showed a maximum of two alleles per locus as expected for a diploid organism. However, for several cases, at least one of the loci showed more than two alleles, and these isolates should thus considered to be aneuploid or polyploid. This complicates the analysis, as most analysis tools assume haploid or diploid data. In previous studies, loci with three alleles were modified to resemble diploid loci by setting one of the alleles to a null allele (Li *et al.* 2010). In this study, alleles were scored in two ways: (i) by assigning specific allele sizes and (ii) by a binary representation of the presence (1) or absence (0) of specific alleles. To avoid the factitious error of heterozygote reduction, a “diploid” dataset was created by including only the minimum and maximum alleles for loci with three stable alleles to assign specific loci (Chen *et al.* 2008). This so-called special diploid dataset was then used to calculate basic measures of genetic diversity described below. The complete binary dataset with “0,” “1” was used to analyze the genetic structure of the Dutch *P. infestans* population as described below.

#### Basic measures of genetic diversity:

To analyze the variation in microsatellite loci, the observed number of alleles (Na), the effective number of alleles (Ne) and Shannon’s information index (I) per locus in all populations were estimated using the special diploid dataset described above with 652 isolates in POPGENE. The significance of deviations from HWE, using Bonferroni corrections, was determined using the special diploid dataset with 358 isolates and exact *P* values estimated by GENEPOP version 4.0 (Raymond and Rousset 1995) and the Markov chain algorithm with 10,000 dememorization steps, 100 batches, and 1000 iterations. GENEPOP 4.0 was also used to calculate the observed heterozygosity (Ho), expected heterozygosity (He), the polymorphism information content (PIC) value, and the level of LD applied to the special diploid dataset with 652 isolates to determine the extent of distortion from independent segregation of loci. To examine the distribution of genetic variation among and within populations, analysis of molecular variance (AMOVA) using Arlequin version 3.5 (Excoffier *et al.* 2005) was employed with the binary dataset of 652 isolates. Arlequin 3.5 was also used to perform two versions of the neutral tests (Ewens-Watterson test and Ewens-Watterson-Slatkin test) to check whether an actual allele frequency deviated significantly from a probability distribution for allele frequencies under the infinite-alleles model in a neutrally evolving population by the special diploid dataset with 652 isolates. BOTTLENECK software version 1.2.02 (Cornuet and Luikart 1996) was employed to test the bottleneck hypothesis under a two-phased model of mutation (TPM) using the same dataset as in Arlequin. BOTTLENECK tests for testing the departure from mutation-drift equilibrium were based on heterozygosity excess or deficiency.

#### Genetic structure analysis:

Two types of clustering methods were used: a model-based method to detect spatial substructures and a genetic distance–based method to explore the diversity by multilocus genotype data (Excoffier and Heckel 2006). The spatial genetic structure was analyzed using the Bayesian clustering program STRUCTURE 2.2 (Pritchard *et al.* 2000) with the binary dataset of 358 isolates. The range for the number of clusters (K) was specified from 1 to 15. For each run, we examined the output for consistency of clustering assignments and checked parameters for convergence. To infer K values and determine the best level of structure supported by the data, a more formal method developed by Evanno *et al.* (2005) calculated the ΔK statistic, the modal value of which can be a useful *ad hoc* indicator for the level of uppermost hierarchical structure (Evanno *et al.* 2005). To perform ΔK calculations, we randomly assigned the likelihood from each of five STRUCTURE runs from each K into one of five groups, each containing a single likelihood from each K. Within each of these five groups, we then calculated the necessary differences from Ln′(K) and |Ln′′(K)|. |Ln′′(K)| was averaged over the five groups, and divided by the standard deviation of the likelihood for the ultimate calculation of ΔK.

To validate the genetic substructure, PCA using NTSYS software (Rohlf 1987, 2008) and the same dataset as in STRUCTURE was conducted to construct plots of the most significant axes for grouping pattern verification.

The genetic distance between individual isolates was calculated using the binary data with all isolates, including the references, PowerMarker software (Liu and Muse 2005), and the neighbor-joining (NJ) method based on the proportion of shared allele distance. PowerMarker is specifically designed to analyze genetic marker data, especially SSR/SNP data. Dendrograms were created by MEGA 4.0 (Tamura *et al.* 2007) using the distance matrix generated by PowerMarker and 1000 bootstrap replications.

### Screening for virulence on potato lines carrying the *Rpi-blb1* gene

A TaqMan PCR was designed to differentiate between *Rpi-blb1* virulent and *Rpi-blb1* avirulent *P. infestans* isolates through amplification of the *Avrblb1*/*ipiO* class I region. *P. infestans* isolates lacking class I *ipiO* variants were shown to be virulent on *Rpi-blb1* (Champouret *et al.* 2009). The PCR was performed in a total volume of 30 µl in an AB mastermix (Applied Biosystems, Veenendaal, the Netherlands) using the 250 nM primers of FW_145 (gaagagcgggcgttttct) and RV_227 (gtcttggactgagtgc) to amplify the *Avrblb1*/*ipiO* class I region and 83.3 nM of the FAM-labeled probe Ipio_1_LNA4 (ctttatgGattcaaACTtgga) to visualize the amplification. As a positive control, the ITS region was amplified using 50 nM FITS1-15ph (tgcggaaggatcattaccacacc) and RITS1-279ph (gcgagcctagacatccactg) visualized by a 83.3 nM of a VIC-labeled probe (cggcTACtgctggc). The PCR profile consisted of 10 min at 95° followed by 40 cycles of alternation between 95° (15 sec) and 60° (60 sec). The specificity of the TaqMan was validated on avirulent isolates T30-4, 90128, 98014, VK1.4, PIC99183, and on virulent isolates PIC99177 and PIC99189, as well as on cloned fragments of the *Avrblb1* gene/class I *ipiO* gene of these isolates.

### Virulence assays on *Rpi-blb1*

Detached leaves of cultivar Desiree and Desiree containing *Rpi-blb1*were placed on 1.5% water agar and spray inoculated with (20,000 sporangia/ml). Leaves were incubated at 15° (16 hr light/8 hr dark regime). After 7 days, the infected leaf area and the presence of spores was evaluated microscopically. If the amount of spores on Desiree containing *Rpi-blb1* was similar to the amount of spores found on Desiree, isolates were scored virulent.

## Results

### Microsatellite polymorphism across loci

In 668 isolates (652 of the 2000–2009 set plus 16 older ones), the 12 microsatellite loci revealed high polymorphism information content (PIC) values ranging between 0.218 and 0.841, with an average value of 0.534 and different allele frequencies (Table 2). G11 is the most informative locus of the 12 SSRs, with the highest value for the Shannon’s information index (I = 2.006). A total of 75 alleles was detected over 12 loci, and the average number of alleles per locus was 6.250, ranging from 3 (Pi4B, Pi70, PinfSSR2, and PinfSSR6) to 17 (D13) alleles per locus. The effective number of alleles, including rare alleles, ranged from 1.155 (Pi70) to 6.151 (G11), with an average of 2.436. Locus D13 had the highest observed number of alleles (17 alleles), but its effective number of alleles (1.872) was under the mean number (2.436) for the whole population. SSR markers revealed 28 rare alleles with a frequency lower than 0.05. The frequency of the rare alleles within loci (Table 2) ranged from 0 (Pi4B, Pi63, PinfSSR6, and PinfSSR11) to 0.429 (D13). Allele sizes ranged from 116 to 356 bp. Locus D13 had the largest range of allele sizes (58 bp), whereas PinfSSR2 and PinfSSR6 had the smallest range (4 bp).

The mean expected heterozygosity (He) was 0.524 (varying between 0.134 and 0.838), and the mean observed heterozygosity(Ho) was 0.577 (varying between 0.114 and 0.809). Five loci were not in HWE. After clone correction, the loci displaying an excess of heterozygotes were still significantly different from HWE (Table 2). The linkage disequilibrium tests revealed linkages between pairs of loci, but these linkages were not consistent across geographic populations. In each population, all loci behaved as neutral according to the Ewens-Watterson test, indicating the absence of clear selection forces influencing the frequency of all SSR loci used in this study.

### Population structure over the temporal and spatial scales

The complete set of isolates showed a high genetic diversity with 322 unique genotypes among 652 field isolates. To examine the distribution of genetic variation among and within populations defined by the five geographical areas, AMOVA was performed. This showed that 95% of the variance could be attributed to the regional stratum (within regions), whereas the remaining 5% was attributed to the national stratum (between regions). This strongly indicates the absence of separate regional populations. In testing the departure from mutation-drift equilibrium based on heterozygosity excess or deficiency, bottleneck analysis was conducted for the regional populations under the two-phased model of mutation of microsatellites. The five geographic populations displayed no significant excess of heterozygosity (*P* > 0.05) through Wilcoxon significant rank test, suggesting that none of these five populations has experienced a recent bottleneck.

**Figure 2:**
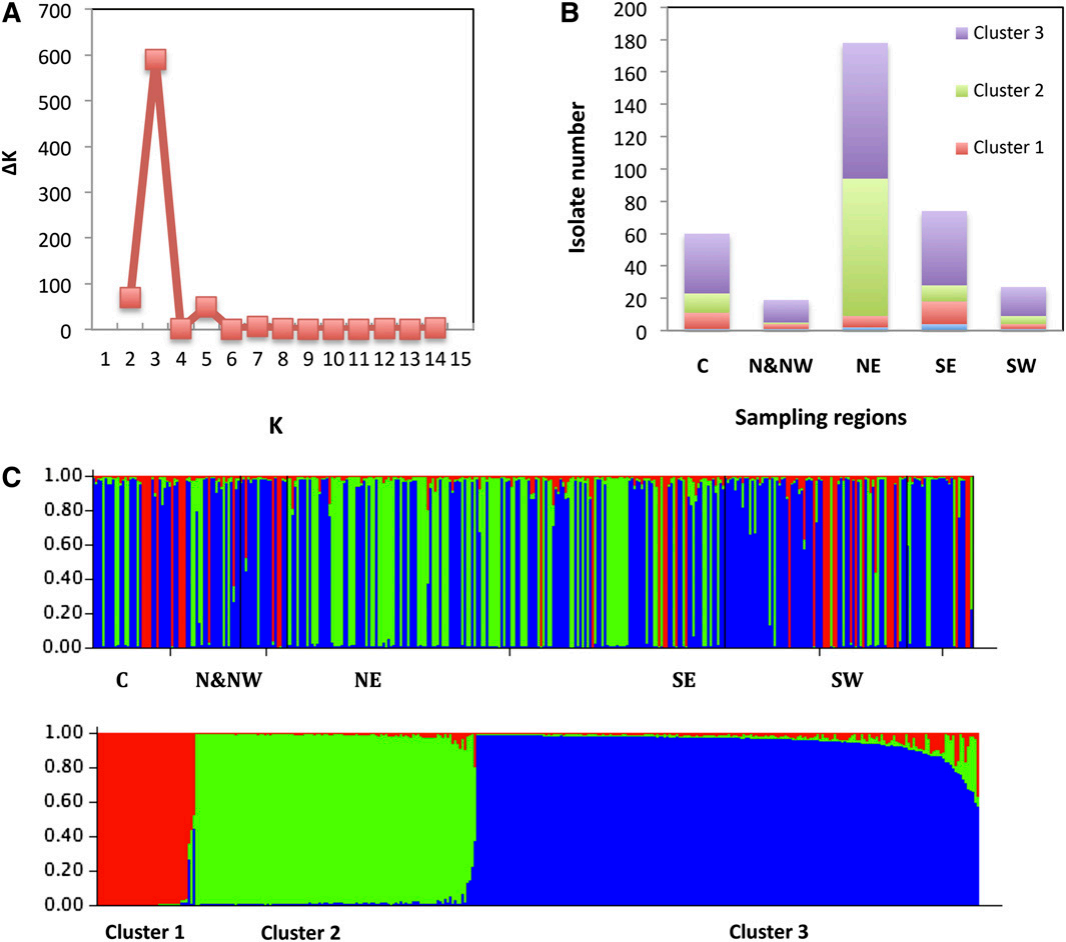
STRUCTURE analysis of 12 microsatellite loci after clone correction. (A) Result of the ΔK calculation, the second-order rate of change of LnP(D) with respect to K (1–15); ΔK for K = 2–14 revealed a single distinct peak at K = 3. (B) Summary of results for STRUCTURE analysis at K = 3 showing the proportion of isolates from five sampling regions. The isolates were assigned in admixture when the membership is below 70%. (C) Plot example of the raw STRUCTURE output for one run (K = 3) organized by geographic region (top) and by Q value.

For STRUCTURE analysis, clone-corrected data were used. Thus, the genetic structure of 358 isolates was analyzed using the correction for STRUCTURE 2.2 outputs as described by Evanno *et al.* (2005) (Figure 2). For all K, memberships were consistent between all runs. The first ΔK peak for K = 3 indicates the presence of three main clusters. When individual isolates with a membership lower than 70% were not taken into account, 9 of 358 isolates were misclassified. The AMOVA for K = 3 indicated that 25% of the variance was attributed to variation between the three clusters and that 75% of the variance was due to variation within clusters. Pair-wise estimates of FST indicated a high degree of differentiation varying from 0.18 between Clusters 2 and 3 to 0.42 between Clusters 1 and 2.

**Figure 3:**
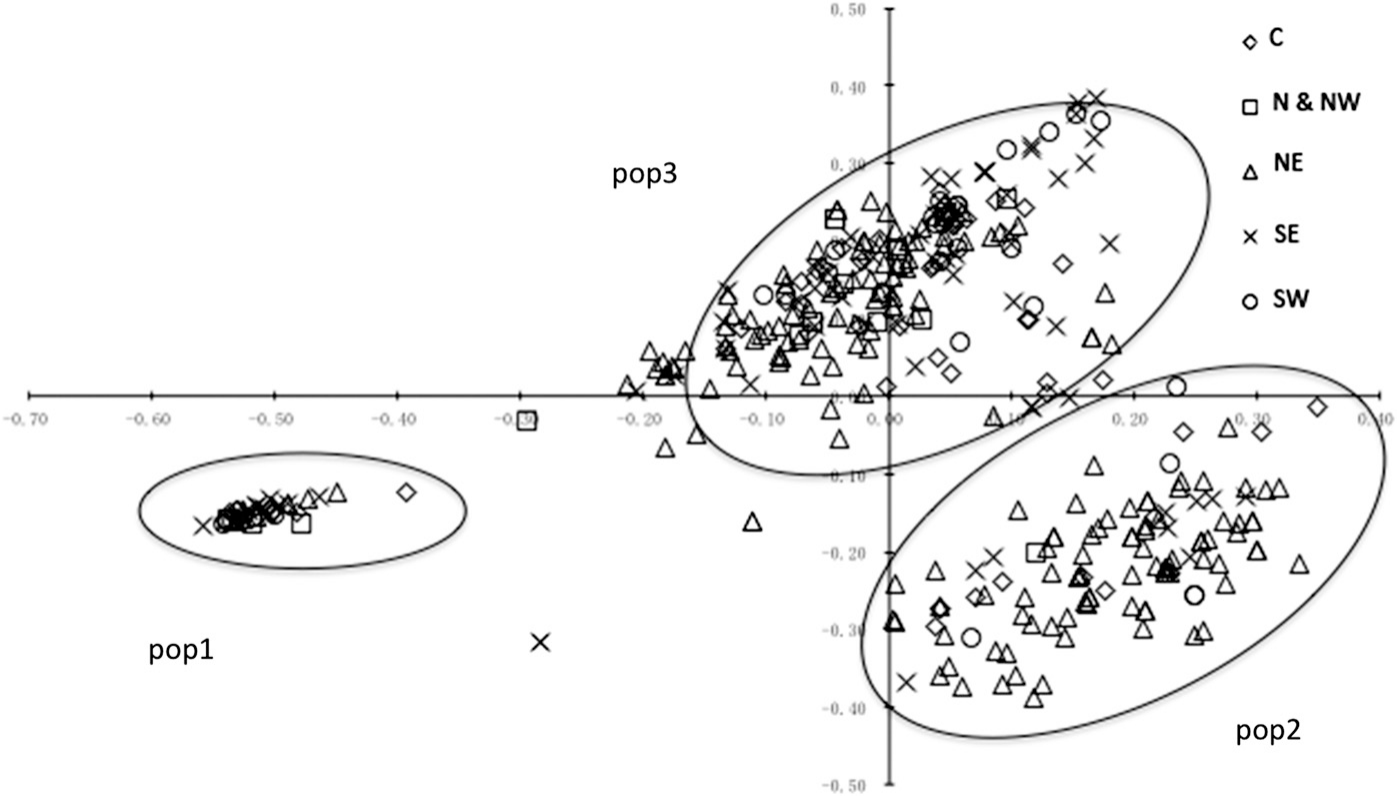
Two-dimensional plot of the first two axes (X = PC1, Y = PC2) of a principal coordinate analysis. Symbols represent the five different sampling regions.

Based on a plot against the first two dimensions from the PCA, three populations were identified (Pop1, Pop2, and Pop3) that correspond to the three clusters identified by the STRUCTURE analysis (Figure 3).

**Figure 4:**
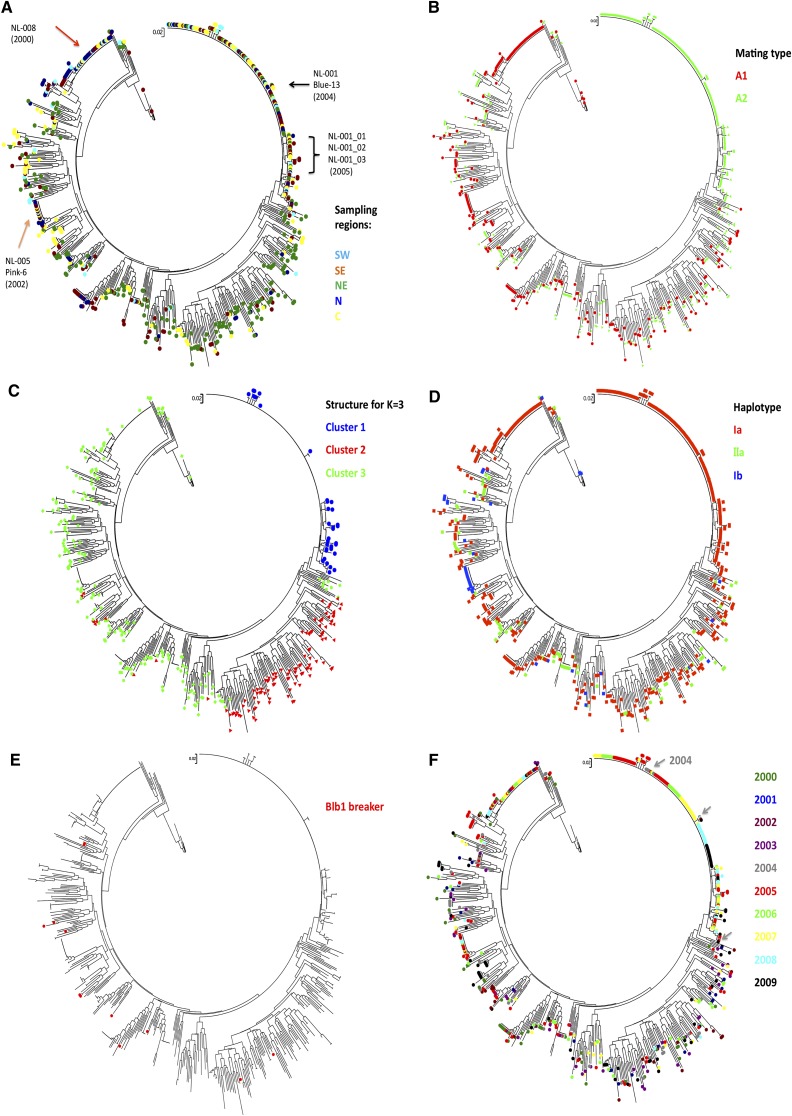
Dendrogram of Dutch *P. infestans* isolates over 10 years using NJ among 12 SSRs. The tree contains 668 isolates and 8 reference isolates (see *Materials and Methods*). The sampling isolates were marked with colors indicating different characters. (A) Five different sampling regions. (B) Mating types. Green indicates the A2 mating type, and red indicates the rest of the isolates identified with the A1 mating. (C) Result of STRUCTURE analysis. For K = 3, three clusters were clearly identified in this figure, and the isolates within three clusters are aligned on the dendrogram. (D) Three haplotypes identified in the Dutch *P. infestans* population. (E) Isolates that lack *Avrblb1/ipiO* class I on the dendrogram. (F) Sampling years of isolates on the dendrogram.

A dendrogram of all 652 isolates from the survey plus the 16 older isolates and 8 SSR reference isolates was constructed using the NJ method (Figure 4, A, B, D, and F). In this tree, isolate VK1.4, a member of the US1 clonal lineage, served as an outgroup. Isolate characteristics, such as geographic origin, mating type, and haplotype, were subsequently superimposed on the dendrogram using different colors (Figure 4, A, B, and D). When the isolate position on the two-dimensional PCA was combined with the dendrogram (Figure 4C), isolates belonging to the NL-001 clade in the dendrogram grouped similarly in Cluster 1 by STRUCTURE and Pop1 by PCA (Figures 2 and 3). Cluster 1 contains only isolates with A2 mating type and mitochondrial haplotype Ia and could be defined as a single clonal lineage with some subclonal variation. Cluster 2 predominantly originates from the North East starch potato region.

Within the three large clusters identified by STRUCTURE, three genotypes are dominant over the various temporal and spatial scales (Figure 4A). One dominant genotype, called NL-001 (A2 and Ia) was retrieved 144 times from isolates collected between 2004 and 2009 covering all five sampling regions and representing 22.1% of the 652 samples. NL-001 has the same SSR genotype profile as the EU13_A2 (or “Blue_13”) clonal lineage previously reported by Lees *et al.* (2009). NL-001_02 (7 isolates), NL-003 (11), and NL-004 (10) are subclonal lineages that show small but consistent differences to NL-001_01 in the dendrogram, and they also have the A2 mating type and Ia haplotype. Out of 12 SSRs, only D13 and SSR4 individually showed two rare alleles in NL-001_02, NL-001_03, and NL-001_04. A second dominant genotype called NL-008 (A1 and Ia) was retrieved 43 times between 2000 and 2009. Genotype NL-005, identical to the previously reported SSR genotype EU6_A1 or “Pink_6” (Cooke *et al.* 2012), grouped 15 isolates collected in five different years (2002, 2005–2008), and it was present in a low frequency (2.3%) over the 10-year period. Next to the three more dominant genotypes, some genotypes were identified at an even lower frequency (less than 1%) with fewer than 15 isolates per genotype, some of which were found in multiple years and multiple regions.

### Spread of dominant clonal lineages

Figure 4F illustrates the temporal dynamics of the genotypes during the years 2000–2009. The vast majority of genotypes were found only once. Isolates with identical SSR genotypes always showed the same mt haplotype and the same mating type, further establishing the clonal identity of these isolates.

The three major clonal lineages (NL-001, NL-005, and NL-008) cover 31% of the entire isolate collection 2000–2009 with 144, 15, and 43 isolates, respectively. Isolates with the NL-008 genotype were found in 2000, the first year of sampling, in all sampling regions except for the NE (Figure 4, A and F). Genotype NL-005 was identified in 2002, and it was found between 2005 and 2008 with a low overall frequency of 2% (Figure 4F). This genotype was retrieved from many regions of the Netherlands from 2002 onwards, but it was not retrieved from the NE.

**Figure 5:**
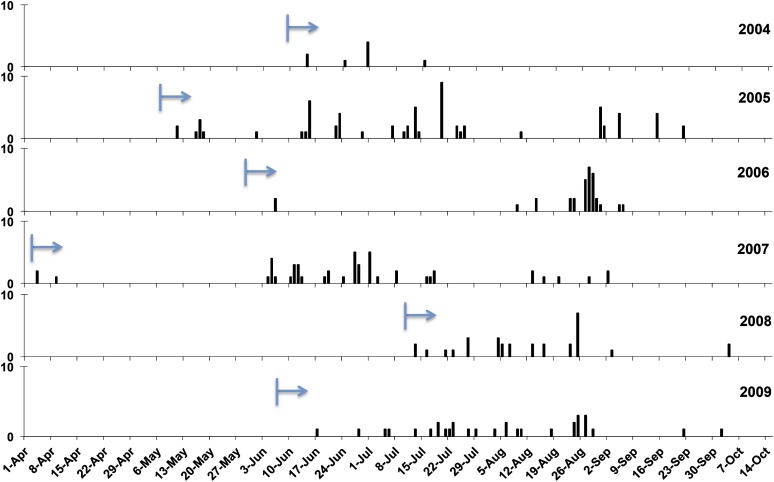
The clonal temporal dynamics of “Blue_13” lineage from 2004 to 2009. The arrow indicates the start time of the first isolate sampling in that season. Vertical bars indicate the day that isolate “Blue_13” was collected, with the height representing the number.

Genotype NL-001 was first found in two regions (NE and SE) in 2004, and it dominated the population from the start (Figure 4F). Since then, NL-001 isolates have been dominantly present in all regions (N & NW in 2009 not sampled) from the very beginning to the very end of every blight season from 2004 to 2009 (Figure 5) with a frequency roughly correlating to the total number of isolates collected in the Netherlands.

### Virulent isolates found in sexual offspring

**Figure 6:**
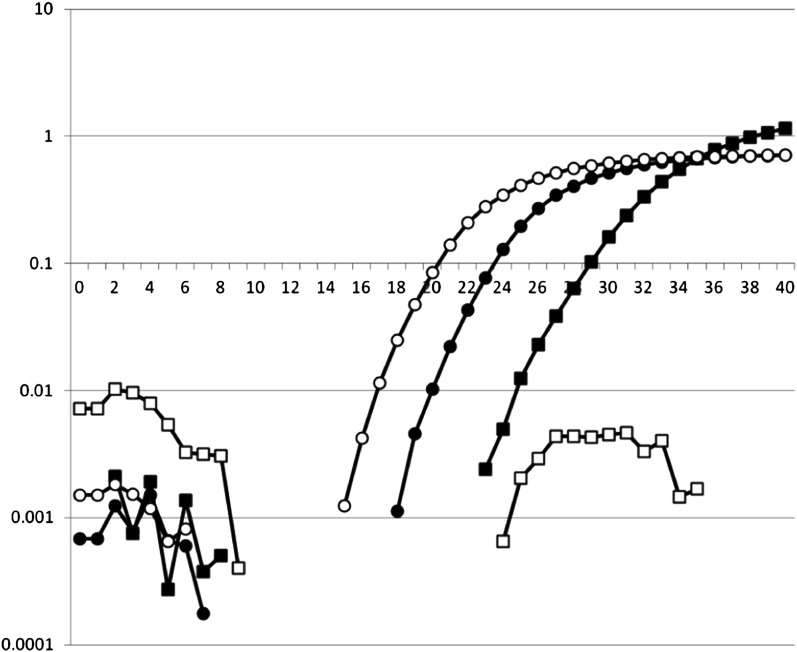
The TaqMan screening for *Avrblb1/ipiO* class I. The number of amplification cycles is shown on the X-axis, and the intensity of detected signal is shown on the Y axis. Open symbols are for the virulent isolate Pic99183, and filled symbols are for avirulent isolate Pic99177. The squares are for *Avrblb1/ipiO* class I, and the circles are for the ITS PCR control. Isolates that show significant increase in the ITS signal but no increase of the *Avrblb1/ipiO* class I signal are virulent.

To determine whether isolates contain *Avrblb1/ipiO* class I, a TaqMan PCR procedure was developed and validated. All *ipiO* class I members (ipiO-01, ipiO-02, ipiO-05, ipiO-07, and ipiO-08) could be detected, whereas for the class II and Class III *ipiO* variants (ipiO-03 and ipiO-04, respectively), no cross-reaction was observed. Combined with the ITS PCR control, this enabled clear differentiation between isolates T30-4, 90128, 98014, VK1.4, and PIC99183, which contain *ipiO* class I variants, and PIC99177 and PIC99189, which do not contain *ipiO* class I variants but do contain *ipiO* class II variants (Figure 6). When the DNA of the field isolates was tested, 12 of the 652 isolate samples (1.8%) lacked an *Avrblb1/ipiO* class I gene. Based on the SSR genotyping, 10 unique genotypes were found among 12 virulent isolates. Isolates with identical SSR genotypes also showed identical results on the *Avrblb1/ipiO* class I screening. No variation for the presence of *Avrblb1/ipiO* class I was found among (subclones of) any of the dominant lineages. Isolates lacking *Avrblb1/ipiO* class I and virulent to *Rpi-blb1*, which was confirmed by bioassay test, were collected in the Netherlands in 2000, 2003–2005, and 2007–2009. Apart from two identical genotypes that will be discussed below, no particular genetic clustering was observed among isolates that lack *Avrblb1* (Figure 4E). In two cases, an identical SSR profile matched with the lack of *Avrblb1/ipiO* class I. Two virulent isolates isolated in 2004 had the same genotype and had originated from the same field and the same potato variety. Two other isolates with an identical genotype were collected in 2005 and 2008. They were sampled in the same region (NE) but from different fields and different potato varieties. Apparently, this genotype was able to survive for several years, but it has not been found since 2008. The other isolates that lack *Avrblb1/ipiO* class I represented unique genotypes, none of which seem to have spread significantly by the asexual cycle. For 11 of the 12 isolates that lack the *Avrblb1/ipiO* class I region, the virulence on *Rpi-blb1* could be tested. All these isolates were virulent on Desiree Rpi-blb1, demonstrating that loss of this region results in virulence.

## Discussion

A better understanding of *P. infestans* population dynamics will contribute to more durable disease management strategies, particularly if high-resolution neutral markers are combined with the use of functional markers, for example, for virulence toward individual *R* genes. Here, we analyzed the Dutch *P. infestans* population over an entire decade, determined the overall population structure, and described the emergence, dynamics, and displacement of the main clonal lineages in correlation with regional cultural practices.

STRUCTURE analysis using a set of 12 highly informative SSR markers splits the Dutch *P. infestans* population into three major clusters, which for Cluster 2, roughly corresponds to the NE geographical region of origin. Cluster 1 consists of a single clonal lineage, NL-001, with 27 subclones previously identified as genotype “Blue_13” (Lees *et al.* 2009). The subclonal lineages showed small but consistent differences, mostly by loss of alleles and by the presence of rare alleles in the SSR loci D13, G11, and PinfSSR4, which are known to be highly variable (Lewontin 1972; Li *et al.* 2010). The clonality of this cluster was further illustrated by the fact that all members have the A2 mating type and the Ia mt haplotype. Also, all members of this cluster have a copy of *Avrblb1/ipiO* class I gene and thus are expected to be avirulent on potato lines carrying the *Rpi-blb1 R* gene. This assumption was confirmed by virulence assays. Members of Cluster 1 were first found in 2004 on multiple locations (NE and SE), and from 2005 on, NL-001 was found in all regions of the Netherlands.

Cluster 2 isolates mainly originate from the NE, a region in which starch potatoes are cultivated almost exclusively. In this region, higher levels of late blight infection are tolerated, especially toward the end of the growing season. Moreover, crop rotation in the NE is generally shorter than in other regions, facilitating oospore-driven epidemics. Corroborating these agronomical practices, oospore-initiated epidemics were reported only in the NE in national surveys in the Netherlands in the period 1999–2005 (Evenhuis *et al.* 2007). In Cluster 2, genetic variation is high and most of the genotypes are found only once (73.0%) or twice (27.0%), the latter often collected from the same field in the same year. This pattern is consistent with a more important role of the sexual cycle and the formation of oospores resulting in high levels of recombination in combination with a limited spread of these genotypes through the asexual cycle. The diversity of this cluster is further illustrated by the absence of clonal lineages and the presence of both mating types (A1 and A2) in a more or less equal ratio (A1/A2 = 161/197) and multiple mt haplotypes (Ia, IIa, and Ib). Some of the isolates of this cluster lack the *Avrblb1/ipiO* class I gene and were found to be virulent on potato plants carrying the *Rpi-blb1* resistance gene.

Cluster 3 includes several (older) clonal lineages in combination with a large number of unique genotypes. The old clonal lineages, such as NL-008 and NL-005 (“Pink_6”), are found in multiple regions and in multiple years, but remarkably, none are found in the NE, despite intense sampling. Cultivars grown in the NE are mainly starch potatoes not grown in the other parts of the Netherlands. Vice versa, cultivars grown in the other parts of the Netherlands are predominantly ware potatoes, including seed production. Furthermore, crop rotation in the NE is every 2–3 years compared with every 4 years in other regions. Shorter crop rotations facilitate oospore-driven epidemics, thus stimulating a more or less local *P. infestans* population in the NE region of the Netherlands. Therefore, the absence of most of the clonal lineages in the NE region may be due to elevated levels of oospore-derived infections masking the infections of the clonal lineages, or it may correlate to differences in resistance of the different cultivars used in this region. These differences are too small to consider the Dutch *P. infestans* population to be a geographically structured meta-population, as supported by the AMOVA analysis on the allele frequencies. However, it can be concluded that the sampled populations reveal a distinct signature for the NE population that could potentially be associated with distinct regional agronomic practices and a slightly more tolerant attitude toward late blight infections in production crops, allowing for increased chances of formation of oospores.

Gene flow can be maintained by dispersal of sexual and/or asexual propagules. In the Dutch *P. infestans* population, re-isolation of clonal isolates was widespread and nationwide. It can only be the result of asexual reproduction. This survey witnesses that the Dutch population underwent dramatic changes in the 10 years under study. The most notable change was the emergence and spread of A2 mating type strain NL-001 or “Blue_13.” Since 2004, NL-001/“Blue_13” has been detected in many countries (Kildea *et al.* 2010), but its origin is unclear (Li *et al*. 2012). The isolates described in this study from 2004 on are the earliest reported for this genotype, which could indicate that NL-001/“Blue_13” originates from the Netherlands. However, there are two remarkable features that may hint to another origin. First, the SSR analysis of these isolates revealed the presence of 27 subclonal variations. Subclonal variants of NL-001 were found even in the first year (2004), and the number of subclonal variants does not show a clear increase over time. Also, in other countries, many subclonal variants were found (Lees *et al.* 2009). This is in sharp contrast with other clonal lineages found in the Netherlands where little or no subclonal variation was found, even though these lineages appear to be present in the Netherlands for a longer period. Thus, it appears that NL-001/“Blue_13” was not introduced into the Netherlands as a single clone but as a set of several subclonal variants. Interestingly, the summer of 2003 had exceptionally high temperatures, which may have resulted in a population bottleneck for the *P. infestans* population and primed the partial displacement. The second remarkable fact that may hint to another origin is the spread of this genotype inside and outside the Netherlands. It was first found in 2004 on multiple locations in the Netherlands, but the genotype was absent in the surveys from 2000 to 2003. It seems unlikely that this dominant genotype would not have been picked up before 2004 if it were present. In addition, NL-001/“Blue_13” was found in multiple locations outside the Netherlands from 2005 on, ranging from various European countries to regions in China (Li *et al.*, unpublished results). It is difficult to envisage how a genotype could spread so rapidly inside and outside the Netherlands, often in the absence of any recorded shipment of (seed) potatoes from the Netherlands.

Although a large part of the Dutch *P. infestans* population has the A1 mating type and the members of the NL-001 clonal lineage generate large amounts of oospores when confronted with the A1 tester in the laboratory mating type test, no clear evidence was found for NL-001/“Blue_13” as parent in the sexual cycle in this study. In an unchanging environment, sexual reproduction of well-adapted genotypes is evolutionarily costly, meaning that most sexual progeny will have a lower fitness than the successful parent. This would promote a strictly asexual strategy until detrimental mutations in a clonal lineage result in reduced fitness.

Some studies indicate that sexual reproduction played a key role in some countries (Brurberg *et al.* 1999; Gavino *et al.* 2000; Nagy *et al.* 2003; Sujkowski *et al.* 1994), whereas in other studies, the contribution of clonality to the population was significant (Blandón-Díaz 2011; Elansky *et al.* 2001; Le *et al.* 2008; Runno-Paurson *et al.* 2009). Although asexual dispersal appears to be important in the species, it was observed that gene flow between the populations depends on the dispersal of the sexual product. Pop2 in the PCA showed a typical pattern of sexual recombination (Figure 3). A previous study suggested that oospores might have acted as an infection source in the Netherlands since the 1990s (Zwankhuizen *et al.* 2000). Our study clearly indicates involvement of an active sexual cycle through the production of oospores. A combined sexual-asexual reproduction/dispersal mechanism seems characteristic for the *P. infestans* population in the Netherlands.

A fast and reliable TaqMan real-time PCR for screening isolates lacking *Avrblb1/ipiO* class I was developed in this study. Previously it was shown that isolates lacking this class of genetic variants are virulent toward *Rpi-blb1* and homologs (Champouret *et al.* 2009). In the Netherlands, virulent isolates were identified, and screening for *Avrblb1/ipiO* classes showed the absence of this class I in these isolates. Application of this TaqMan real-time PCR on the Dutch *P. infestans* isolates (data not shown) yielded several additional isolates lacking the *Avrblb1/ipiO* class I variant. Virulence bioassays demonstrated that also these isolates were virulent on potato lines carrying the *Rpi-blb1* gene. This is the first time that such isolates are reported to occur outside Mexico. Superimposing the virulence screening on the SSR genetic backbone shows that lack of the *Avrblb1/ipiO* class I variant occurred in 12 isolates, representing 10 unique genotypes that do not show any particular genetic association. In most cases, these unique genotypes did not demonstrate successful dispersal through the asexual cycle. In all but one case, the specific genotype was not retrieved in following years. The one case where the genotype managed to survive for several years may be of special interest. Isolates with this genotype should be studied in more detail for phenotypic traits as it may have compensatory mutations or recombined genes compensating the loss of *Avrblb1/ipiO* class I gene. None of the clones and subclones of the clonal lineages showed lack of *Avrblb1*, indicating the region is stable during mitosis. The lack of recombination between variants of *ipiO* class I, *ipiO* class II, and *ipiO* class III (Champouret *et al.* 2009) suggests that these classes are not allelic. Therefore, isolates that lack *ipiO* class I variants may have lost this region, possibly during meiosis. In meiosis, translocation can lead to null alleles as can nondisjunction previously found to occur in *P. infestans* (Carter *et al.* 1999; van der Lee *et al.* 2004). In this study, the first *Rpi-blb1* breakers were found in 2000, long before this gene was cloned (Song *et al.* 2003; van der Vossen *et al.* 2003). Until today, no varieties have been released with this particular *R* gene. Therefore, the occurrence of isolates that lack the *Avrblb1/ipiO* class I variant could be regarded as a random genetic “accident” beneficial in case potato lines with *Rpi-blb1* or its homologs are grown. Bouwmeester *et al.* (2011) recently reported that the *ipiO* gene may be important for virulence, so the reduced spread of genotypes that lack the *Avrblb1/ipiO* class I gene could be in line with this. Nevertheless, the lack of the spread of the virulent isolates observed should be treated with care, as selection pressure was mostly absent and most of the other sexual progeny do not spread and seem to be lost in next year as well. Fortunately, the frequency of virulence toward the *Rpi-blb1 R* gene in the Dutch *P. infestans* population is still low. Therefore *Rpi-blb1* and similar *R* genes, particularly in combination, may provide effective protection in an integrated pest management strategy where monitoring of the *P. infestans* population for virulence and preventive fungicide is applied during periods of high disease pressure. Also, preventive action toward the sexual cycle could be of key importance to the durable introduction of the *Rpi-blb1* resistance gene and possibly other *R* genes.

## Supplementary Material

Supporting Information
