# Supplementary material for: Population Dynamics of Phytophthora infestans in the Netherlands Reveals Expansion and Spread of Dominant Clonal Lineages and Virulence in Sexual Offspring
Source: G3 (Bethesda). 2012 Dec 1;2(12):1529–40. doi: 10.1534/g3.112.004150 (PMC3516475; doi:10.1534/g3.112.004150)
Supplement: Supporting Information [file supp_2_12_1529__index.html]

Supporting Information 

# Population Dynamics of *Phytophthora infestans* in the Netherlands Reveals Expansion and Spread of Dominant Clonal Lineages and Virulence in Sexual Offspring

## Supporting Information for Li *et al.*, 2012

**Files in this Data Supplement:**

- File S1 - Supporting data (.xlsx, 53 KB)
